# Langerhans Cell Histiocytosis Presenting as Progressively Worsening Neck Pain: A Case Report With a Review of Literature

**DOI:** 10.1177/2324709619886757

**Published:** 2019-11-06

**Authors:** Nithya Krishnan, Lara Zuberi, Amal Shukri, Arun Gopinath, Dalys Haymes

**Affiliations:** 1 University of Florida, Jacksonville, FL, USA

**Keywords:** Langerhans cell histiocytosis, histiocytosis X, rare childhood diseases, neck mass

## Abstract

A 29-year-old female with past medical history of chronic serous otitis media presented with worsening neck stiffness and pain over a period of 2 weeks. The patient described non-specific symptoms that were localized to the right side of her neck. She presented to the hospital only when the pain was so extreme that it limited her range of motion. The differential for acute neck pain without fever, chills or any inciting trauma is vast. They include medical emergencies such as meningitis, acute coronary syndromes and extend to rheumatologic diseases or simply musculoskeletal strain. On review of systems, she denied dizziness, headache, vision changes, dysphagia, or other facial pain. Based on the severity of her pain, she underwent a Computed Tomography scan of the neck, which was concerning for erosive calavarial lesions. Further imaging revealed multiple lytic foci and erosions from the right maxillary sinus to the right mandible to the C1 vertebra. Following requisite surgical intervention, she was found to have Langerhans cell histiocytosis, a rare disease of myeloid cells, usually affecting pediatric populations. Little is known about the adult manifestations of Langerhans Cell Histiocytosis. This review contributes to broadening the literature on this topic which can present with complaints as typical as neck pain.

## Case Report

In this article, we report a case of a 29-year-old female who presented with worsening complaints of neck stiffness and pain over a period of 2 weeks. The patient recounted that the pain and tightness was only on the right side of her neck, radiating to the back of her head. It was worse with movement and progressed to the point where the pain limited her range of motion, causing her to present to the hospital. Shortly before this, she had been diagnosed with chronic serous otitis media associated with hearing loss, for which she had tympanostomy tubes placed. Following the surgery, she developed malodorous drainage from the ear, which continued up to her current presentation, despite treatment with otic drops. Her past medical history was unremarkable. She denied dizziness, headache, vision changes, dysphagia, or other facial pain.

**Figure 1.**
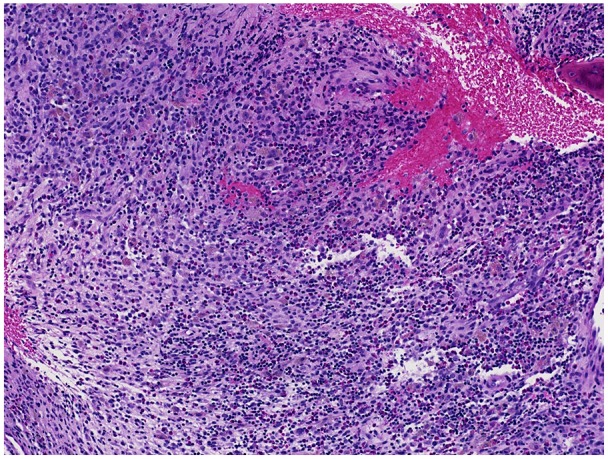
Bone, seen at top right, involved by sheets of mixed inflammatory cells composed of histiocyte, eosinophils, lymphocytes, and plasma cells.

**Figure 2.**
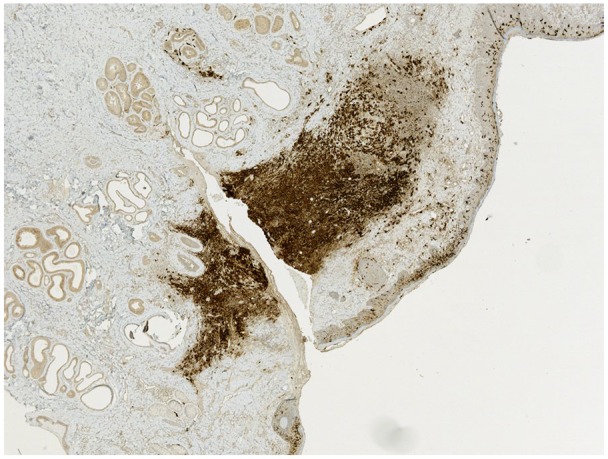
Immunohistochemical stain preparation showing diffuse CD1a expression in the histiocytes (brown color).

**Figure 3.**
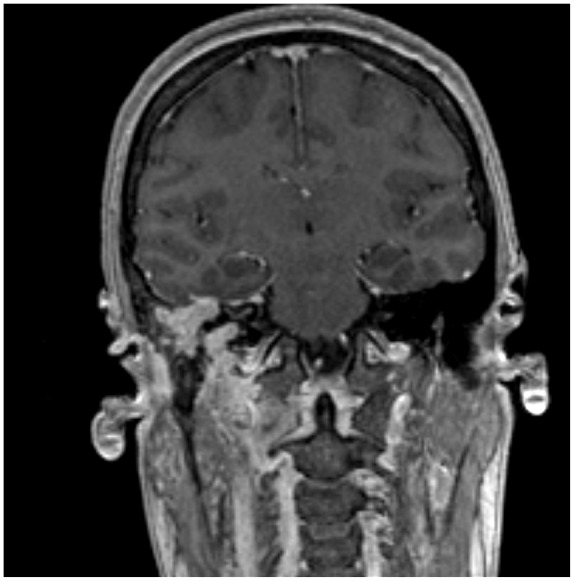
Coronal post-contrast magnetic resonance imaging shows homogenously enhancing soft tissue mass in the right mastoid air cells, extending along the tegmen tympani, associated with reactive dural thickening.

**Figure 4.**
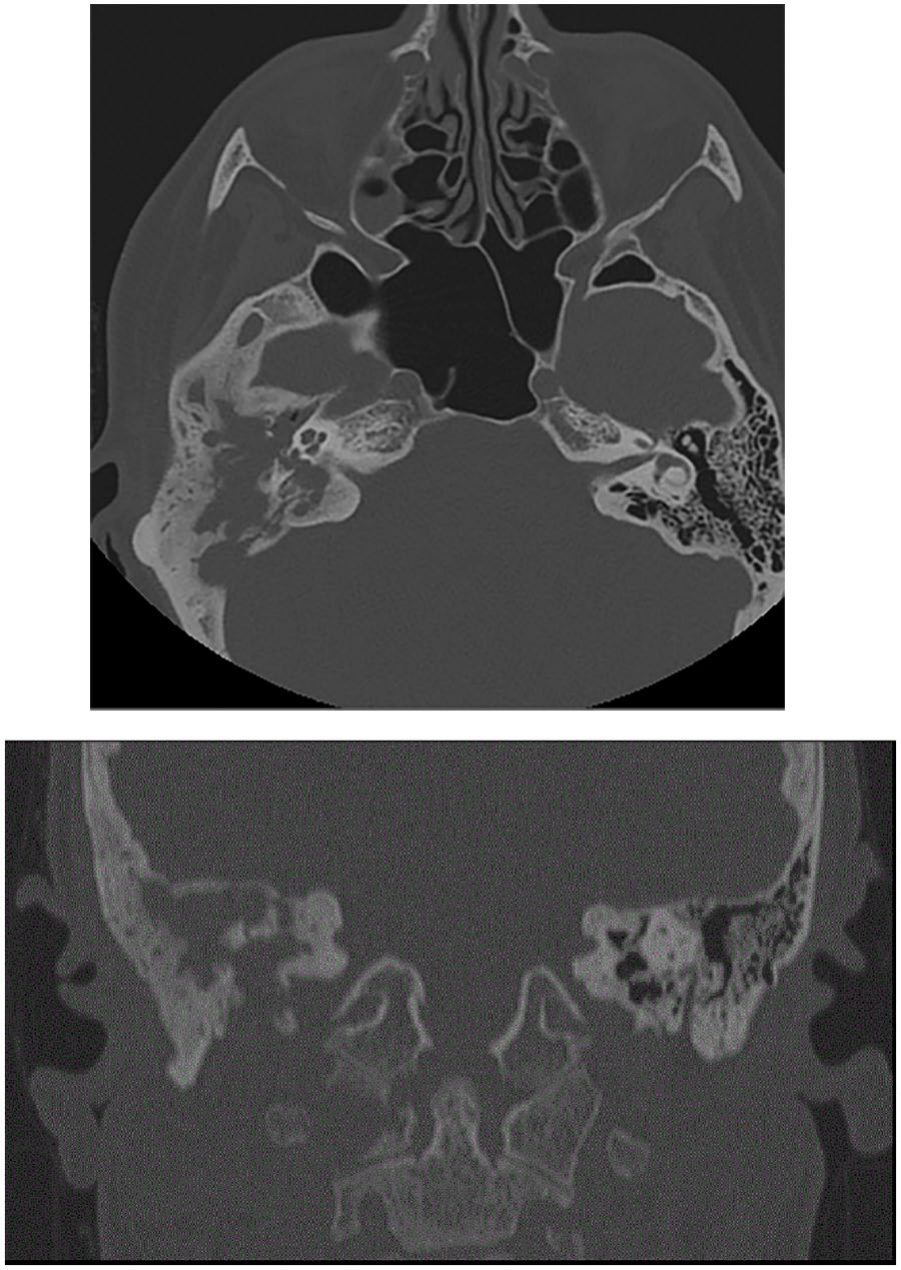
Axial and coronal computed tomography image shows opacification and calescent destructive changes of right mastoid air cells, erosions of right tegmen, middle ear ossicles, and sigmoid plate as well as osteolytic lesion in right lateral mass of C1.

The initial computed tomography scan was concerning for acute right mastoiditis, with erosion of the skull and the first cervical vertebra. Neurosurgery and ENT (ear, nose, and throat) were consulted at that time for further recommendations. A subsequent magnetic resonance imaging brain and C-spine showed mass-like enhancement of the tegmen tympani and reactive dural changes. There were also multiple lytic foci present in the right temporal bone, first cervical vertebral body, and posterolateral wall of the right maxillary sinus wall and mandible. She was taken for surgery, where a right mastoidectomy with right middle ear exploration was performed. Surgical pathology samples returned positive for histiocyte proliferation in a background of eosinophilic and lymphoplasmacytic inflammation consistent with Langerhans cell histiocytosis (LCH). Immunohistochemical stains performed demonstrated diffuse positivity for CD1a and S-100. Further staining was positive for Langerin (CD207). After evaluation by hematology oncology, the patient underwent skeletal survey and pan-computed tomography scan to assess for systemic disease. There was no evidence of lung, liver, or spleen abnormalities. Given her presentation with involvement of craniofacial bones, she was deemed to be high risk of central nervous system involvement. Thus, she was offered outpatient treatment with systemic chemotherapy with prednisone versus vinblastine/prednisone (see Figures 1-4).

## Immunophenotyping

Histiocytic disorders can be characterized by surface markers. Correlating with the clinical picture, the histologic and immunophenotypic characteristic of this lesion is consistent with LCH due to positive staining for CD1a, S-100, and CD207 (Langerin).^1^

Immunophenotyping in LCH can be complicated, as myeloid dendritic cells express CD1a and CD207 as do skin Langerhans cells. In contrast, other histiocytic disorders such as Erdheim-Chester or juvenile xanthogranulomatous disease are thought to be derived from dendritic cells producing a monocyte-macrophage lineage and therefore not sharing the same immunophenotype. The markers more characteristic of the macrophage lineage include CD14/CD68/CD163 and have been observed to be more aggressive with a higher mitotic index.^1,2^

## Genomic Testing

The advent of genomic testing has allowed for increased specificity of identifying LCH. The activating mutations in the proto-oncogene BRAF-V600E have been implicated in several cancers including melanoma and colorectal cancer. In LCH, 65% of patients have had the BRAF V600E mutation in pathologic dendritic cells. This can be used to further support a diagnosis of LCH, though it is not part of risk stratification directing therapeutic intervention. Some studies show that the presence of the gene correlates with high-risk clinical features, higher risk of relapse, and more aggressive disease.^3^

## Chemotherapeutics

Current standard-of-care for initial chemotherapy is vinblastine/prednisone in patients who require systemic treatment. Other therapies include observation or prednisone therapy alone. Patients who fail initial chemotherapy may require salvage therapies including cytarabine, cladribine, and clofarabine. These salvage treatments have resulted in very high rates of cure among high-risk patients, but were associated with prolonged hospitalization and treatment-related death. There still remains a lack of consensus on treatment, but this is mostly due to the unclear pathophysiology of LCH, which acts both as a neoplastic disorder and an immunodysregulatory disorder.^1^ There have been several trials over the past decades that have shown improvement in outcomes; however, high-risk patients (those with risk of central nervous system involvement) have <50% chance of progression-free survival. These data are mainly applicable to the pediatric population, as research on adult treatment outcomes is ongoing.^1^ Yet, it has been shown that in those individuals who experience treatment failure or relapse, there is an increased long-term risk of complications including neurodegeneration. Data from the European registry from 2000 to 2013 show a 3-year overall survival rate of 71% to 77% with allogenic hematopoietic-cell transplantation, which may also be an option for refractory LCH.^2^ In adults, LCH may present as mixed-phenotype lesions, coexisting with other myeloid neoplasms. In general, adults have a less robust response to chemotherapy than children. Vemurafenib is a BRAF V600E inhibitor that has been studied at length. The VE-BASKET trial reported metabolic responses assessed by positron emission tomography scans in all patients. A report including 8 patients with LCH who underwent treatment with vemurafenib noted relapse after discontinuing the medication. The optimal use of BRAF inhibitors in adjunct to standard chemotherapy remains under study. Another drug currently under investigation is cobimetinib, an MEK-inhibitor, which can be used in patients who may or may not express the BRAF mutation.^4^

## Discussion

Though exceedingly rare, this case highlights the importance of broad differentials when working up common complaints such as radicular neck pain in a young patient without history of trauma. LCH is a rare disease most commonly characterized by osteolytic bone lesions. Epidermal Langerhans cells are dendritic cells, a heterogeneous group of hematopoietic cells. The pathophysiology, while unclear, has now been shown to activate somatic MAPK mutations in myeloid precursor cells, which is how LCH has come to be considered a myeloid neoplastic disorder, though there are immunoregulatory factors also at play.^1^ On pathology, bone lesions show infiltration with histiocytes—polygonal cells with eosinophilic cytoplasm, oval nuclei with longitudinal grooves resembling coffee beans. In addition to bone, these histiocytes, along with macrophages, lymphocytes, and eosinophils, may infiltrate soft tissue, most often skin, lungs, liver, and spleen. LCH is most commonly reported in children from ages 1 to 3 years, with the incidence in adults being miniscule, approximately 0.0002%.^5^ Advances in LCH research have informed classification of LCH as a disease of myeloid differentiation; however, now the task remains to translate the biologic advances into improved clinical outcomes in adult patients.
